# An Algorithm for the Noninvasive and Personalized Measurement of Microvascular Blood Viscosity Using Physiological Parameters

**DOI:** 10.1155/2020/7013212

**Published:** 2020-08-31

**Authors:** Ge Sun, Lin Yang, Weiwei Wang, Song Zhang, Zhichang Luo, Guanghui Wu, Xiaohong Liu, Dongmei Hao, Yimin Yang, Xuwen Li

**Affiliations:** ^1^Faculty of Environment and Life Sciences, Beijing University of Technology, Beijing 100124, China; ^2^Beijing International Science and Technology Cooperation Base for Intelligent Physiological Measurement and Clinical Transformation, Beijing 100124, China; ^3^Fuwai Hospital, Chinese Academy of Medical Sciences, Beijing 100037, China; ^4^Beijing Institute of Heart Lung and Blood Vessel Diseases, Beijing Anzhen Hospital, Capital Medical University, Beijing 100029, China; ^5^Beijing Yes Medical Devices Co. Ltd., Beijing 100152, China

## Abstract

Blood viscosity is one of the important parameters to characterize hemorheological properties of the human body. Its real-time and dynamic measurement has important physiological significance for studying the development and prevention of chronic diseases. This study researched noninvasive and personalized measurement of microvascular blood viscosity. In the microcirculation capillary network blood flow model, combined with pulse wave parameters, multiple regression analysis was used to fit the simulated radius of personalized physiological blood vessels to calculate the microvascular blood viscosity. The fitted value related to the simulated radius of the physiological blood vessel had a high correlation with the corresponding theoretically derived value (correlation coefficient: 0.904, *P* ≤ 0.001). The calculated value of the microvascular blood viscosity had a certain correlation with the clinical whole blood viscosity at a low shear rate (correlation coefficient: 0.443, *P* < 0.05). This algorithm could provide effective means for noninvasive and long-term individual monitoring and family health care.

## 1. Introduction

Hypertension, hyperglycemia, and hyperlipidemia have become high-risk factors for cardiovascular diseases such as coronary heart disease, atherosclerosis, and myocardial infarction [1–6]. The application of automatic household detectors for blood pressure and blood glucose on the market is very common. Generally, blood lipids can only be checked by the blood tests in hospitals. Hyperlipidemia and other diseases normally have a blood viscosity abnormality syndrome before the obvious symptoms appeared [7–9]. Blood viscosity can be evaluated based on whether an individual is in an abnormal state of blood viscosity, thereby providing auxiliary means for monitoring of hyperlipidemia. Blood viscosity is the internal friction generated between the various molecules inside the blood when it flows in the blood vessels. It characterizes the condition of blood circulation or supply [10, 11]. In different pathological conditions, the change law of blood viscosity can also provide useful data for the diagnosis, treatment, and prognosis of the disease [12–14].

There have been two main methods for detecting microvascular viscosity: invasive and noninvasive. The invasive detection method was mainly performed by collecting human anterior cubital vein blood. The blood viscosity measurement methods commonly used have been trauma and in vitro detection, such as capillary viscometer and rotational viscometer [15]. This type of method mainly used instruments to measure the friction between molecules in the blood to calculate the blood viscosity value, which was a clinical “gold standard.” Blood viscosity decreases with increasing shear rate. Clinically, the whole blood viscosity under a high shear rate was detected by setting the shear rate of the rotational viscometer at 200 s^−1^. It mainly characterized the state of blood flow in the large blood vessels. The blood flow state of blood in microvessels was mainly characterized by the viscosity of whole blood under a low shear rate. It was clinically detected by setting the shear rate of the rotational viscometer at 1 s^−1^. Noninvasive detection methods were mainly implemented by biomechanical modeling or waveform feature extraction based on the pulse wave.

The current invasive and noninvasive detection methods for blood viscosity had certain defects. Invasive operation was complicated and professional. Therefore, it was difficult to realize a continuous and dynamic blood viscosity detection function for individuals. The algorithm of noninvasive detection technology based on biomechanical modeling was complicated and inaccurate. It was difficult to transplant the mobile terminal platform of the algorithm. The waveform feature extraction method was a noninvasive detection technology based on pulse wave hemodynamics. This method mainly collected and analyzed the human pulse wave waveform to extract the characteristic parameters of the waveform. Then, the blood viscosity algorithm could be established according to the correlation between the waveform characteristic parameters and the blood viscosity to obtain the human blood viscosity value [16]. This method based on the pulse wave did not consider the influence of factors such as the individual differences, the radius of the blood vessel, and the ability of the heart to supply blood on blood viscosity. The test results had certain limitations.

Research on noninvasive detection of blood viscosity based on pulse wave has accumulated certain results and experience. However, in terms of theoretical research, there was still a lack of analysis of blood viscosity in vivo and comprehensive research of transplantable blood viscosity models. This study combined pulse wave parameters to study noninvasive and personalized measurements of microvascular blood viscosity. It was beneficial to further improve the enthusiasm for continuous monitoring of blood viscosity in patients with chronic diseases.

## 2. Materials and Methods

### 2.1. Subjects and Specimens

The subjects of this study were 79 male patients with cardiovascular and cerebrovascular diseases in the Beijing Anzhen Hospital of the Capital Medical University from 2013 to 2014. The 79 samples of the study had complete records of epidemiological examination, hemorheology examination, biochemical index examination, echocardiography, and other examination results. The basic data information is shown in Table 1.

This study was fully approved by the local Ethics Committee, Beijing International Science and Technology Cooperation Base for Intelligent Physiological Measurement and Clinical Transformation.

The blood flow characteristics reflected by the blood viscosity of whole blood under different shear rates are different. As the shear rate decreases, it indicates that the blood flow rate in the blood vessel is slower. In microvessels, the flow velocity of the blood is only about 10^−1^ cm/s, which is a blood flow state at a low shear rate [11]. The gold standard for the corresponding blood viscosity measurement in clinical practice is the blood viscosity of the whole blood under a low shear rate. The low shear rate clinically characterized the low-speed blood flow state when the shear rate was 1 s^−1^ [15]. Although this blood flow rate was different from the blood flow rate in microvessels proposed in this study, it was of the same order of magnitude. The clinically detected whole blood viscosity at a low shear rate value was taken as the actual value, and the calculated microvascular blood viscosity value based on this study was compared with it. Based on the gold standard for clinical blood viscosity detection, the blood flow conditions of microvessels in people with different degrees of obesity were studied, establishing the model and algorithm to calculate blood viscosity for noninvasive and personalized measurement.

In this study, SPSS Statistical Analysis Software (SPSS Inc. SPSS for Windows 13.0. Chicago, Illinois, USA) was used for statistical analysis.

### 2.2. Algorithm Establishment

#### 2.2.1. Proposal of the Microcirculation Capillary Network Blood Flow Model

This study was based on pulse wave hemodynamic noninvasive detection technology and selected the human fingertip photoplethysmography (PPG) signal that was highly repetitive and easy to operate individually [17]. Various waveform parameters of the PPG signal are one of the effective methods to observe and evaluate the state of the microcirculation [18, 19]. The basic structure of the microcirculation is the microvessels. This method is simple and easy to operate and is suitable for noninvasive measurement of microvascular blood viscosity. This research is aimed at establishing a blood viscosity model based on the flow characteristics of blood in microvessels to achieve noninvasive detection of blood viscosity.

Based on the above reasons, the microcirculation capillary network blood flow model was proposed to simulate the blood flow state of the blood in human microvessels. According to the characteristics and functions of microvascular blood flow, it was assumed that the radial gap between microvessels and the side branches of each microvascular inlet segment was ignored. Then, the complex capillary network of the human body was simulated as a slender round tube model with radius *R* and length *l*.

Blood is a type of non-Newtonian fluid [20]. But in this model, if the blood flowed at a specific shear rate, it could be regarded as a Newtonian fluid. In microvessels, blood flows slowly and the shear rate is low. In the case of the fixed low shear rate, the blood of the microvessels could be regarded as a Newtonian fluid. The flow characteristics of the blood in microvessels meet the conditions of this model. At this time, the blood in microvessels made steady laminar flow in the model to meet the flow conditions of Poiseuille's law [21, 22].

#### 2.2.2. Derivation of the Human Microvascular Blood Viscosity Calculation Equation

The prerequisite for this study was that the blood in microvessels met Poiseuille's flow. Simultaneously, the law of capillary substance exchange was satisfied. Combined with the microcirculation capillary network blood flow model, the blood viscosity calculation equation of the microvessels meeting the above prerequisites could be derived.

According to Poiseuille's law [21, 22] and the above model conditions, the blood viscosity value “*μ*” could be calculated by Equation (1) (unit: cp).

(1) $$\begin{array}{c} \mu =\frac{\pi \cdot R^{4}\cdot \Delta p}{8\cdot Q\cdot l}. \end{array}$$

In Poiseuille's flow law of Newtonian fluids, calculating fluid viscosity involves the following parameters: volumetric flow of the fluid during laminar flow in a horizontal uniform circular tube: *Q* (unit: cm^3^/s), pressure difference between the two ends of the tube: Δ*p* (unit: mmHg), tube radius: *R*, and tube length: *l* (unit: cm). Each parameter unit could be converted according to calculation needs.

However, all the parameters in the Equation (1) represented the actual blood flow and blood vessel values of the human body, and they were not easy to measure. In order to facilitate the noninvasive detection and calculation of microvascular blood viscosity, according to the microcirculation hemodynamic characteristics and pulse wave related theory, various formal parameters in the Equation (1) were converted into practical parameters with clinical significance.

Corresponding to the model above-mentioned, the parameters for calculating the microvascular blood viscosity were derived as follows.

*(1) Volume Flow: Q*. According to Equation (2), *Q* refers to the product of the average velocity per unit time and the cross-section area perpendicular to the axial direction of the circular tube.

(2) $$\begin{array}{c} Q=u_{m}\cdot \pi \cdot R^{2}, \end{array}$$

where *u*_*m*_ (unit: cm/s) is the average blood flow velocity in the circular tube of the model and *R* is the simulated radius of the circular tube.

*(2) Pressure Difference between the Two Ends of the Tube: Δp*. As blood flowed through systemic blood vessels, blood viscosity encountered resistance, causing blood pressure in the blood vessels to drop. During the entire circulation flow, the pressure drop caused by blood flowing through the aorta and middle arteries only accounted for about 10% of the total systemic pressure drop. Relative to the arterioles and capillaries, the pressure drop caused by the aorta and middle arteries could be ignored. Therefore, when measuring the pressure difference between the two ends of the circular tube in the model, the pulse pressure difference PP measured by the human brachial artery could be used instead.

*(3) Round Tube Simulation Inside Diameter: R*. The actual capillary diameter of the human body is only in the order of 10^−5^, and the unit is m. Due to the large number and complexity of capillaries, its cross-sectional area is on the order of 10^−1^, and the unit is m^2^. If each capillary was closely aligned along the same axis, the human microvascular network was simulated as an aggregated capillary. It could be simulated as a slender round tube with the radius *R* and the length *l*. Then, the calculated blood vessel radius was in the order of 10^−2^, and the unit was m.

The true blood vessel radius of the human kidney capillary bed could not be measured [23]. *R* is the simulated radius of the model's physiological blood vessels, and there is no direct measurement method. In this study, the population was distinguished by the body mass index (BMI) international standard, and then the clinical data were linearly fitted to obtain the fitting equation.

*(4) Tube Length: l*. Based on the assumption that blood flowed in a round tube, it was expressed by the product of average blood flow velocity and blood retention time, as shown in the following equation:

(3) $$\begin{array}{c} l_{m}=u_{m}\cdot t_{m}. \end{array}$$

*t*_*m*_is the average residence time of blood in the capillary bed (unit: s).

According to the clinical physiological test report, the calculation method of the total blood flow *Q* through the capillary model per unit time was as described:

(4) $$\begin{array}{c} Q=\frac{\text{CO}}{60}. \end{array}$$

CO is cardiac output (unit: cm^3^/min). CO was calculated by the following equation:

(5) $$\begin{array}{c} \text{CO}=\text{SV}\cdot \text{HR}. \end{array}$$

SV is the stroke volume (unit: ml/beat) and HR is the heart rate (unit: beat/min). SV was calculated by Equation (6) [24].

(6) $$\begin{array}{c} \text{SV}=\frac{\left\{\left(7\cdot {R_{\text{CLD}}}^{3}/\left(2.4+R_{\text{CLD}}\right)\right)-\left(7\cdot {R_{\text{CLS}}}^{3}/\left(2.4+R_{\text{CLS}}\right)\right)\right\}}{100}. \end{array}$$

*R*_CLD_ is the left ventricular end diastolic diameter (unit: mm) and *R*_CLS_ is the left ventricular end systolic diameter (unit: mm).

In the microcirculation capillary network blood flow model, the average residence time *t*_*m*_ of the blood flow is the inverse of the half-update rate ALK of the blood flow [16]. The average retention time *t*_*m*_ of blood flow and the half-update rate of blood flow ALK were calculated as shown in Equations (7) and (8) below [16].

(7) $$\begin{array}{c} t_{m}=\frac{1}{\text{ALK}}, \end{array}$$

(8) $$\begin{array}{c} \text{ALK}=25.2\times 10^{-3}\times \frac{\text{CO}}{\text{BSA}}. \end{array}$$

As shown in Equation (9), BSA [25] is the body surface area (unit: m^2^).

(9) $$\begin{array}{c} \text{BSA}=0.0061\times H+0.0128\times W-0.1592. \end{array}$$

*H* is the height (unit: cm), and *W* is the weight (unit: kg).

According to the above Equations (1) to (9) and the derivation of related parameters, the blood viscosity calculation Equation (10) containing clinically measurable physiological parameters could be obtained.

(10) $$\begin{array}{c} \mu =\frac{14904\times R^{6}\times \text{PP}}{\text{BSA}\times \text{CO}}. \end{array}$$

The physiological parameters included in the Equation (10) are PP, BSA, CO, and *R*. *R* was obtained through clinical data statistics.

### 2.3. Statistical Analyses

In the model mentioned above, the multiple regression analysis method in SPSS 13.0 software was used. The *R*-related linear fitting was performed on the crowd of different BMI classification, and the fitting equation was obtained to calculate the estimated microvascular blood viscosity.

In order to verify the accuracy of the algorithm and the corresponding model results, this study used Pearson correlation analysis in SPSS 13.0 software. If the significance level *α* = 0.05 and the statistical index *P* < 0.05, the correlation will be significant. Generally, the value of correlation coefficient describes the degree and direction of the linear correlation between the two variables. The absolute value of correlation coefficient is between 0 and 1. A larger value indicates a stronger correlation.

## 3. Results

### 3.1. Calculation of the Simulated Radius of the Physiological Blood Vessel and Corresponding Blood Viscosity

Among all model parameters, PP, BSA, and CO could be calculated from clinical data. However, the simulated radius of the physiological vessels in the model was a formal parameter and could not be obtained from clinical detection. For people with different physical characteristics, the actual number and diameter of microvessels are variable. This study used the BMI international standard to personalize the normal and relatively overweight population. Corresponding simulated radius fitting equations were established to effectively distinguish individual differences.

BMI was calculated by Equation (11), and its international standard is shown in Table 2.

(11) $$\begin{array}{c} \text{BMI}=\frac{W}{H^{2}}. \end{array}$$

*W* represented weight (unit: kg) and *H* represented height (unit: m).

The analysis of this study was based on the WHO standards. The selection criteria were mainly due to the data composition of this study, as shown in Table 3.

One of the 79 cases had a large deviation value of pulse pressure difference, so this data was excluded. The 78 cases of data in this study were randomly divided into two groups, the experimental group and the validation group. There were 40 cases of data in the experimental group and 38 cases of data in the validation group, almost 1 : 1. After the experimental group and the validation group were grouped, the variance of the BMI and other parameters of the above groups was verified, to ensure that the two groups did not introduce errors between groups during the grouping process.

On the premise that the Equation (10) proposed in this paper was valid, the calculation Equation (12) of *R*^6^ was obtained.

(12) $$\begin{array}{c} R^{6}=\frac{\mu \times \text{BSA}\times \text{CO}}{14904\times \text{PP}}. \end{array}$$

*R* ^6^ was the calculated value obtained from clinical data and was used as the fitted object. Multivariate regression analysis method was used to linearly fit the population grouped by different BMI classification, and the fitting equations were shown as follows:

(13) $$\begin{array}{c} {R^{6}}_{\text{BMI}=1}=‐0.334+0.196\times \frac{\text{SV}}{\text{PP}}+0.004\times \text{HR}, \end{array}$$

(14) $$\begin{array}{c} {R^{6}}_{\text{BMI}=0}=‐0.18+0.151\times \frac{\text{SV}}{\text{PP}}+0.003\times W. \end{array}$$

According to the “overweight criteria” in Table 2, the corresponding subscript BMI in Equations (13) and (14) was expressed as 1 and 0, respectively. Then, personalized blood viscosity “*μ*′” calculation was conducted, as shown in the following:

(15) $$\begin{array}{c} \mu^{\prime }=\frac{14904\times {R^{6}}_{\text{BMI}}\times \text{PP}}{\text{BSA}\times \text{CO}}. \end{array}$$

### 3.2. Correlation Calculation Verification

The simulated radius of the physiological blood vessel and the accuracy of the final blood viscosity calculation equation were verified as follows.

Pearson correlation analysis was used to calculate the correlation coefficient between *R*^6^_BMI_ and *R*^6^ and the correlation coefficient between *μ*′ and *μ*, as shown in Table 4. At this time, *μ* represented the measured value of the clinical whole blood viscosity at a low shear rate.

**Figure 1:**
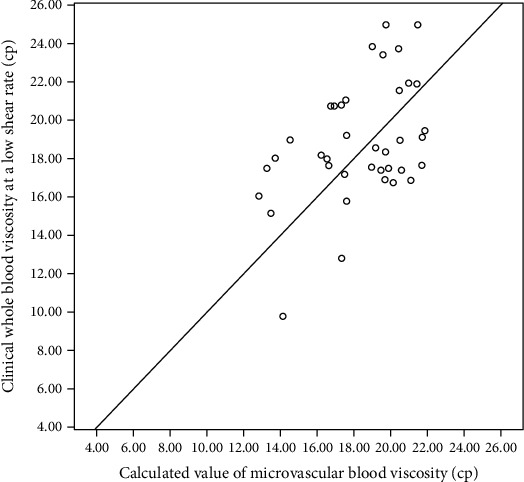
Comparison of calculated and measured microvascular blood viscosity.

Personalizing the calculation first, the overall trend could be judged and verified. As shown in Table 4 and Figure 1, the correlation between the calculated microvascular blood viscosity in this study and the clinically detected blood viscosity at a low shear rate was 0.443 (*P* < 0.05). *P* < 0.05 proved that the calculated value of this study was significantly related to the actual value of clinical detection.

To a certain extent, the blood viscosity value calculated in this study based on the microcirculation capillary network blood flow model could represent the clinical whole blood viscosity at a low shear rate.

## 4. Discussion

Ionescu and Clara established a memory model based on the mathematical calculus for blood viscosity but did not verify it [26]. Kaliviotis and Yianneskis proposed a method based on a viscoelastic model to describe the mechanical energy of a fluid volume and reflect the blood viscosity value through the rate of change of energy [27]. This model performed better when the shear rate was above 0.1 s^−1^, and the model sensitivity of the shear rate at 0.277 s^−1^ was 0.42. This method established a blood viscosity model from the perspective of micro hemodynamics and could describe the dynamic blood viscosity value in vivo. However, it was mainly aimed at the study of the difference in blood viscosity of a particular disease, which had certain limitations and did not have a strong correspondence with clinical blood viscosity value. In 2019, Horner et al. improved the Horner-Armstrong-Wagner-Beris (HAWB) model and increased the viscoelastic equation of red blood cells in the blood to simulate transient hemorheology [28]. Although it had potential application value, the calculation was complicated and it was not easy for individual monitoring. In the study of blood viscosity measurement models, Luo et al. found that the pulse contour characteristic value *K* had a good correlation with blood viscosity [16]. Although the blood viscosity detection method was simple to calculate, it did not consider the influence of factors such as individualized differences in the detection object and the state of blood vessels on blood viscosity.

This study corresponded to the clinical whole blood viscosity at a low shear rate and studied the blood viscosity value in vivo. Combining a simple model, algorithm, and convenient pulse wave signal acquisition method, a noninvasive and personalized measurement of microvascular blood viscosity could be achieved. Simultaneously, the algorithm of this study improved the accuracy of noninvasive detection of clinical whole blood viscosity at a low shear rate.

## 5. Conclusions

Pulse wave parameters and a microcirculation capillary network blood flow model were combined to study noninvasive and personalized measurements of microvascular blood viscosity. Multivariate regression analysis was used to fit the personalized simulated radius of the physiological blood vessels to calculate the blood viscosity of the microvessels. The Pearson correlation analysis was performed for accuracy verification. The algorithm based on the noninvasive approach was simple and effective. It could provide certain reference values for real-time, dynamic, and long-term monitoring of individual cardiovascular conditions, especially for the research and prevention of chronic diseases.

Due to the limitation of the amount of data in this study, no further algorithmic research was carried out to distinguish between personalized differences. In the future, it is necessary to continue to refine different people and expand the sample size.

## Figures and Tables

**Table 1:** Basic data structure.

	Quantity	Mean ± SD
Basic parameters		
Age	79	59.05 ± 8.72
Pulse rate (beat/min)	79	72.10 ± 7.50
BMI	79	25.95 ± 3.15
DBP (mmHg)	79	74.16 ± 10.15
SBP (mmHg)	79	128.59 ± 15.04
Height (cm)	79	168.80 ± 5.87
Weight (kg)	79	74.11 ± 10.84
SV (ml/beat)	79	92.21 ± 21.28
End diastolic vessel diameter (mm)	79	50.27 ± 5.73
End systolic vessel diameter (mm)	79	34.12 ± 6.36
Model parameters		
BSA (m^2^)	79	1.82 ± 0.16
CO (ml/min)	79	6636.71 ± 1642.29
PP (mmHg)	79	54.43 ± 11.83

Notes: BMI: body mass index; DBP: diastolic blood pressure; SBP: systolic blood pressure; SV: stroke volume; BSA: body surface area; CO: cardiac output; PP: pulse pressure.

**Table 2:** BMI international standard.

BMI classification	WHO standards
Thin	<18.5
Normal	18.5-24.9
Overweight	≥25
Preobese	25.0-29.9
Obese	30.0-34.9
Severe obesity	35.0-39.9

**Table 3:** The data structure based on BMI international standard.

	Experimental group		Validation group	
	Normal	Overweight	Normal	Overweight
Number	18	22	17	21
Mean ± SD	23.13 ± 1.61	27.94 ± 2.35	23.33 ± 1.23	27.95 ± 1.87

**Table 4:** Correlation coefficient between parameters.

	*R* ^6^_BMI_		*μ*	
	*r*	*P*	*r*	*P*
*R* ^6^	0.904	≤0.001	—	
*μ*′	—		0.443	0.005

## Data Availability

The raw data required to reproduce these findings cannot be shared at this time as the data also forms part of an ongoing study.
